# Perceived Impact of Spasticity Is Associated with Spatial and Temporal Parameters of Gait in Multiple Sclerosis

**DOI:** 10.5402/2012/675431

**Published:** 2012-01-17

**Authors:** Swathi Balantrapu, Brian M. Sandroff, Jacob J. Sosnoff, Robert W. Motl

**Affiliations:** Department of Kinesiology and Community Health, University of Illinois at Urbana-Champaign, Urbana, IL 61801, USA

## Abstract

*Background*. Spasticity is prevalent and disabling in persons with multiple sclerosis (MS), and the development of the Multiple Sclerosis Spasticity Scale-88 (MSSS-88) provides an opportunity for examining the perceived impact of spasticity and its association with gait in this population. *Purpose*. This study examined the association between the perceived impact of spasticity and spatio-temporal parameters of gait in persons with MS. *Methods*. The sample included 44 adults with MS who completed the MSSS-88 and 4 walking trials on a 26-foot GAITRite^TM^ electronic walkway for measurement of spatio-temporal components of gait including velocity, cadence, base of support, step time, single support, double support, and swing phase. *Results*. The overall MSSS-88 score was significantly associated with velocity (*r* = −0.371), cadence (*r* = −0.306), base of support (*r* = 0.357), step time (*r* = 0.305), single leg support (*r* = −0.388), double leg support (*r* = 0.379), and swing phase (*r* = −0.386). *Conclusions*. The perceived impact of spasticity coincides with alterations of the spatio-temporal parameters of gait in MS. This indicates that subsequent interventions might target a decrease in spasticity or its perceived impact as an approach for improving mobility in MS.

## 1. Introduction

Multiple sclerosis (MS) is an inflammatory, neurodegenerative disease of the central nervous system (CNS). The disease process itself results in the demyelination and transection of axons in the brain, spinal cord, and optic nerves [1]. This axonal damage delays or blocks the propagation of electrical potentials along neuronal pathways in the CNS [2] and the extent and location of damage manifests in the heterogeneous expression of symptoms [3].

Spasticity defined as the velocity-dependent increase in the muscle stretch reflex, and exaggerated tendon jerks resulting from hyperactivity of motor components to sensory input [4] is a common, disabling, and potentially self-limiting symptom of MS that requires a patient-focused, multidisciplinary approach for management [5]. Spasticity itself affects upwards of 90% of persons with MS [6, 7], typically presents in the lower legs (i.e., calf muscles), and has been associated with worse mobility disability [7–9]. To date, there is limited information on the patient's perceived impact of spasticity and its association with temporal and spatial parameters of gait that are targets of gait rehabilitation in MS. This is important as perceptions of the impact of spasticity might represent a target of therapeutic interventions for gait rehabilitation. 

Recognizing the importance of capturing the perceived impact of spasticity for its eventual management, researchers undertook a systematic development and comprehensive psychometric evaluation of the Multiple Sclerosis Spasticity Scale-88 (MSSS-88) [10]. The MSSS-88 emerged as a reliable and valid patient-reported outcome of the perceived burden of spasticity for impacting a patient's life. This scale has the potential for advancing our understanding of the perceived impact of spasticity in clinical trials and practice involving persons with MS. Such an opportunity is important as spasticity management should be patient-focused and account for the impact of a patient's experiences and perceptions of spasticity in impacting everyday life activity such as walking mobility. To that end, one first step for actualizing this potential involves examining the association between MSSS-88 scores and temporal and spatial parameters of gait.

This laboratory-based study examined the association between the patient's perceived impact of spasticity and temporal and spatial markers of gait using MSSS-88 scores and the GAITRite^TM^ electronic walkway, respectively. The GAITRite^TM^ electronic walkway provides a rapid, clinically relevant measurement of spatio-temporal gait components including velocity, cadence, base of support, step time, single and double support, and swing phase and has been validated in MS [11, 12]. The present examination is important for identifying the possible association between the patient's perception of spasticity and gait itself—an important component of everyday life function. We further note that this examination was motivated by the possibility that patient-focused, clinical trials and practice might target the perception of spasticity and its impact as a means of improving mobility in MS. We expected that the perception of spasticity would be associated with worse spatial and temporal characteristics of gait in persons with MS such that MSSS-88 scores would be positively correlated with base of support, step time, and double leg support and negatively correlated with cadence, velocity, single leg support, and swing phase.

## 2. Methods

### 2.1. Participants and Procedures

The procedure was approved by an Institutional Review Board, and participants provided appropriately obtained written informed consent. We recruited a sample of persons with MS through direct contact with support groups of a Midwestern chapter of the National Multiple Sclerosis Society that were located within an approximately 90-minute drive from our campus. The inclusion criteria involved a clinically definitive diagnosis of MS, relapse-free during the past 30 days, ambulatory, 18–64 years of age, and having the visual ability necessary to read 14-point font. We screened 69 persons with MS and 44 were enrolled in the study. On a single testing session, the participants provided demographic and clinical information, completed the MSSS-88, and performed 4 trials of walking on the GAITRite^TM^ electronic walkway for measuring spatial and temporal parameters of gait.

### 2.2. Primary Measures

#### 2.2.1. MSSS-88

The MSSS-88 is a self-reported measure of the burden of spasticity in persons with MS [10]. This scale has 88 items that are rated on a 4-point scale of 1 (Not at all bothered) through 4 (Extremely bothered). The items correspond with 8 subscales of Muscle Stiffness, Pain and Discomfort, Muscle Spasms, Activities of Daily Living, Walking, Body Movements, Emotional Health, and Social Functioning. The overall measure of spasticity burden was computed by averaging the mean scores for the 8 subscales on the MSSS-88 [13].

#### 2.2.2. GAITRite

Participants completed 4 trials of walking on a 26-foot GAITRite^TM^ (CIR systems, Inc) electronic walkway at a comfortable pace as done in previous research involving persons with MS [11, 12]. The GAITRite^TM^ system is a computerized instrument with sensors arranged in a gridlike pattern for identifying footfall contacts. The system provides cadence, stride length, step time, base of support, and percentages of gait cycle spent in double support, single support, and swing phase as spatial and temporal measures of gait. We recorded the cadence (steps/min), velocity (cm/sec), step time (sec), base of support (cm), single leg support (%), double leg support (%), and swing phase (%) for each of the 4 trials. The average of the 4 trials for each variable was used in the analysis for improved reliability.

#### 2.2.3. PDDS

The Patient Determined Disease Steps (PDDS) is a self-report scale that measures walking mobility using an ordinal scale of 0 (Normal) through 8 (Bedridden). This scale was developed as an inexpensive surrogate for the Expanded Disability Status Scale (EDSS) and scores from the PDDS are linearly and strongly related with physician-administered EDSS scores (*r* = .93) [14].

### 2.3. Data Analysis

Descriptive and inferential data analyses were conducted using SPSS version 18 (SPSS Inc, Chicago, Illinois). We first provided descriptive statistics including mean, standard deviation (SD), and range of scores for the MSSS-88 and spatial and temporal parameters from the GAITRite^TM^. We then provided inferential statistics for the relationship between MSSS-88 scores and the spatial and temporal parameters of gait based on bivariate correlation analysis using Pearson product-moment correlation coefficients (*r*). The alpha-value for statistical significance was 0.05.

## 3. Results

### 3.1. Sample Characteristics

The study participants were ambulatory women (*n* = 38) and men (*n* = 6) with a definite diagnosis of MS. The mean ± SD age of the sample was 47 ± 9 years, and the sample primarily had relapsing-remitting MS (*n* = 38; 86%) with a mean ± SD disease duration of 11 ± 8 years and a median PDDS score of 1 (Interquartile Range (IQR) = 0 and 3).

### 3.2. Descriptive Statistics

#### 3.2.1. Spasticity

MSSS-88 data from this study are reported in Table 1. The mean MSSS-88 score in the present study was lower than that reported in a previous study [13] of ~1.9. This is expected because that previous study recruited participants with spasticity based on modified Ashworth Scale (MAS) scores between 1 and 3.

#### 3.2.2. Spatial and Temporal Parameters

Data for the spatial and temporal parameters of gait are reported in Table 2. The sample had a faster cadence, step time, and velocity when compared with a previous study [11]. This difference is expected and explained by the lower level of disability in the present study compared with the previous study [11].

### 3.3. Bivariate Correlation Analysis

**Figure 1:**
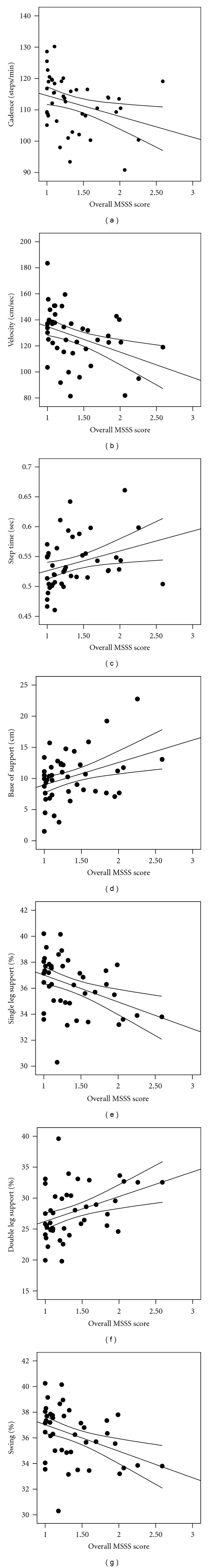
Scatter plot, linear trend line, and 90% confidence intervals for the association between MSSS-88 and cadence (steps/min), velocity (cm/sec), step time (sec), base of support (cm), single leg support (%), double support (%), and swing phase (%).

The correlations between overall MSSS-88 scores with spatial and temporal parameters of gait are provided in Table 3. Scatter plots of the associations between MSSS-88 and cadence, velocity, step time, base of support, single leg support, double leg support, and swing phase are provided in Figure 1. Collectively, the overall MSSS-88 score was significantly correlated with gait components: velocity (*r* = −0.371, *P* = 0.007), cadence (*r* = −0.306, *P* = 0.022), base of support (*r* = 0.357; *P* = 0.009), step time (*r* = 0.305; *P* = 0.022), single leg support (*r* = −0.388, *P* = 0.005), double leg support (*r* = 0.379, *P* = 0.006), and swing phase (*r* = −0.386, *P* = 0.005).

There further were significant associations between MSSS-88 subscales and spatial and temporal parameters of gait as reported in Table 3. The strongest pattern of correlations emerged for the perceived muscular burden of spasticity, whereas the weakest pattern of correlation existed for the feeling or emotional burden of spasticity.

## 4. Discussion

The novel finding of the present study was that the perceived impact of spasticity was associated with spatial and temporal parameters of gait. Those who reported a greater overall burden of spasticity had worse spatial and temporal parameters of gait, namely, reduced cadence, velocity, single leg support, and swing phase as well as increased step time, base of support, and double leg support. The subscales of the MSSS-88 were further associated with the parameters of gait. This extends previous research reporting that clinical measures of spasticity are associated with worse performance on mobility outcome measures in persons with MS [7, 9, 15]. Collectively, this body of research indicates that spasticity and its perceived impact are associated with worse mobility, and our data further indicate that the perception of spasticity and its burden might represent an important target of gait rehabilitation as gait is the physical manifestation of walking.

 Our study is different from previous study [9, 15] in that we measured the patient's perspective on spasticity and its impact on gait kinematics, whereas previous studies included clinical methods of measuring spasticity and its impact on ambulatory performance. For example, one study of persons with MS measured spasticity by the MAS and reported that worse leg spasticity was associated with worse walking speed, endurance, and balance indicated by worse performance on T25FW, 6-Minute Walk Test (6MWT), and Berg Balance Scale, respectively [9]. An additional study measured spasticity based on the amplitude of the soleus H-reflex (electrophysiological marker of spasticity) and reported that it was associated with postural sway while on a force platform [15]. By comparison, the present study primarily focused on the perceived impact of spasticity and gait kinematics (i.e., the physical basis of walking) and reported that MSSS-88 scores were associated with spatial and temporal parameters of gait. Collectively, this indicates that the manifestation and perception of spasticity are associated with worse mobility, including gait kinematics, in persons with MS.

One possible implication of our research is that the perceived impact of spasticity represents an important target of therapies for improving mobility and gait in MS. Whereas many therapies have been developed for targeting spasticity itself (e.g., pharmacotherapy, muscle stretching, whole-body vibration), there is very limited information on approaches for targeting the perceived impact of spasticity in MS. Exercise training might represent a possible approach for reducing the perceived impact of spasticity in MS. Indeed, one study adopted a quasi-experimental design and reported that a 4-week period of unloaded leg-cycling exercise was effective for reducing MSSS-88 scores compared with a control condition, but the exercise training did not change electrophysiological and clinical markers of spasticity in persons with MS [13]. This is similar with other research indicating that exercise training is effective for reducing the perceived impact of spasticity based on MSSS-88 scores in persons with MS [16, 17]. Beyond exercise training, self-management strategies might be an additional route for reducing the perceived impact of spasticity in MS. Such strategies include gaining knowledge about factors that can trigger spasticity including postural change, diurnal variations, incomplete emptying of bladder/bowel, pressure sores, tight clothing, emotions, and stress [18]. There are likely to be other strategies for reducing the perceived impact of spasticity in MS, and a critical step in such efforts will involve examinations of the secondary effects on walking performance and gait kinematics, particularly as some therapies that are effective for reducing spasticity can have negative effects like muscle weakness, fatigue, and paresthesia [19].

Overall, this laboratory-based, cross-sectional study provides novel evidence that overall perceived impact of spasticity coincides with alterations in spatial and temporal parameters of gait in persons with MS. This highlights the importance of considering the patient's perspective regarding the impact of spasticity as a target of an intervention for improving mobility and gait kinematics in MS. Collectively, additional research is required that identifies methods and approaches for managing the perceived impact of spasticity as this holds promise to be a significant component of improving mobility and gait in persons with MS who have spasticity. 

## Figures and Tables

**Table 1:** Mean, standard deviation and range of subscales.

	Mean ± SD	Range
MSSS_overall	1.4 ± 0.4	1–2.6
MSSS_muscle	1.5 ± 0.5	1–2.9
MSSS_pain	1.5 ± 0.6	1–3.4
MSSS_spasms	1.2 ± 0.3	1–2.2
MSSS_activity	1.2 ± 0.4	1–3.0
MSSS_walking	1.6 ± 0.7	1–3.3
MSSS_body	1.3 ± 0.5	1–2.7
MSSS_feeling	1.3 ± 0.5	1–2.8
MSSS_social	1.3 ± 0.5	1–2.5

MSSS: Multiple Sclerosis Spasticity Scale.

**Table 2:** Mean, standard deviation, and range of spatial and temporal parameters of gait.

	Mean ± SD	Range	Mean (Previous study results)
Cadence (steps/min)	112 ± 8	90–130	94.4

Velocity (cm/sec)	127.8 ± 20.9	81.4–183.5	85.5

Step time (sec)	0.5 ± 0.04	0.5–0.7	0.67(R)
			0.65(L)

Base of support (cm)	10.3 ± 4	1.5–22.8	11.6(R)
			11.6(L)

Single leg support (%)	36.3 ± 2.1	30.3–40.2	37.3(R)
			36.7(L)

Double leg support (%)	27.7 ± 4.2	19.8–39.6	24.6(R)
			24.9(L)

Swing phase (%)	36.3 ± 2.1	30.3–40.3	39.1(R)
			39.8(L)

Previous study results [11].

**Table 3:** Bivariate correlations among MSSS-88 components and gait parameters during walking on a GAIRite^TM^ system.

	Cadence (steps/min)	Velocity (cm/sec)	Step time (sec)	Base of support (cm)	Single leg support (%)	Double leg support (%)	Swing phase (%)
Overall MSSS-88	−0.31*	−0.37*	0.31*	0.36*	−0.39	0.38*	−0.39
MSSS-Muscle	−0.32*	−0.35*	0.31*	0.35*	−0.39	0.38*	−0.39
MSSS- Pain	−0.20	−0.32*	0.19	0.39	−0.32*	0.31*	−0.32*
MSSS-Spasm	−0.23	−0.32*	0.23	0.40	−0.40	0.39	−0.40
MSSS-Activity	−0.20	−0.32*	0.21	0.17	−0.36*	0.35*	−0.36*
MSSS-Walking	−0.31*	−0.35*	0.31*	0.39	−0.26	0.25	−0.26
MSSS- Body	−0.30	−0.31*	0.29	0.39	−0.39	0.39	−0.39
MSSS-Feeling	−0.20	−0.24	0.21	0.11	−0.24	0.22	−0.23
MSSS-Social	−0.25	−0.25	0.26	0.11	−0.30*	0.29	−0.30

*Correlation is significant at the 0.05 level (2-tailed).

MSSS: Multiple Sclerosis Spasticity Scale.
